# The prevalence and correlates of undiagnosed HIV among Australian gay and bisexual men: results of a national, community-based, bio-behavioural survey

**DOI:** 10.7448/IAS.18.1.20526

**Published:** 2015-11-11

**Authors:** Martin Holt, Toby Lea, Jason Asselin, Margaret Hellard, Garrett Prestage, David Wilson, John de Wit, Mark Stoové

**Affiliations:** 1 Centre for Social Research in Health, UNSW Australia, Sydney, Australia; 2 The Burnet Institute, Melbourne, Australia; 3 Infectious Disease Unit, The Alfred Hospital, Melbourne, Australia; 4 Department of Epidemiology and Preventive Medicine, Monash University, Melbourne, Australia; 5 The Kirby Institute, UNSW Australia, Sydney, Australia

**Keywords:** Australia, bio-behavioural survey, gay and bisexual men, undiagnosed HIV, prevalence, risk factors

## Abstract

**Introduction:**

Gay and bisexual men (GBM) with undiagnosed HIV are believed to contribute disproportionately to HIV transmission in Australia but national prevalence estimates have been lacking.

**Methods:**

From November 2013 to November 2014, we recruited men at gay venues and events in six Australian states and territories. Of 7291 survey participants, 3071 men also provided an oral fluid sample for testing and decided whether to receive their test results or not. We calculated raw and population-weighted prevalence estimates and identified associations with undiagnosed infection using logistic regression.

**Results:**

Of 3071 participants, 213 men tested HIV-positive (6.9%, 95% confidence interval [CI] 6.0 to 7.8%), of whom 19 (8.9%, 95% CI 5.8 to 13.5%) were previously undiagnosed. After weighting for the size of the gay and bisexual male population in each state or territory, national HIV prevalence was estimated to be 7.2% (95% CI 6.3 to 8.1), of which 9.1% (95% CI 6.0 to 13.6%) were estimated to be undiagnosed. Compared with HIV-negative participants, men with undiagnosed HIV were more likely to report meeting partners at sex venues, using antiretroviral drugs as pre-exposure prophylaxis, condomless anal intercourse with casual partners, using party drugs for sex, injecting drugs and using amyl nitrite, crystal methamphetamine or gamma hydroxybutyrate in the six months prior to the survey.

**Discussion:**

The results indicate that the prevalence of undiagnosed HIV is relatively low among Australian GBM but is higher among men who report riskier sex and drug practices.

**Conclusions:**

The results underline the importance of targeted HIV prevention and frequent testing for men at increased risk of infection.

## Introduction

Gay and bisexual men (GBM) remain the population group most affected by HIV in Australia, accounting for 79% of all HIV infections since the epidemic began and 70% of recent diagnoses [1]. By the mid-2000s, approximately 5% of Australian GBM were believed to be HIV positive [2,3], while more recent behavioural surveillance in metropolitan areas has found that up to 10% of GBM report being HIV positive [4,5]. However, the direct measurement of HIV prevalence and undiagnosed infection among Australian GBM is rare. Anonymous prevalence studies of GBM attending gay bars and sex venues were conducted in Queensland in 2007 and Victoria in 2008 [6,7]. These studies found that 9 to 10% of participants were HIV positive and 20 to 31% of HIV-positive GBM were unaware of their infection. Modelling of the Australian HIV epidemic has suggested that 9 to 12% of GBM with HIV are undiagnosed [8–10]. No prevalence studies have been conducted before in Australian cities with notable GBM populations like Sydney, Adelaide or Perth [2].

Studies in high-income countries with concentrated epidemics among GBM find widely varying levels of HIV prevalence and undiagnosed infection. Based on anonymous studies that recruited GBM from gay venues and events from 2003 to 2011, HIV prevalence has been found to range from 3 to 17% in Europe to 18 to 19% in Canada and the United States [11–15]. The levels of undiagnosed infection among HIV-positive GBM also vary, ranging from 14% in Canada to over 40% in some western European cities [11–15]. In San Francisco, where there has been an intense focus on increasing access to HIV testing and treatment, HIV prevalence among GBM remained stable at 23 to 24% during 2004 to 2011 while the proportion of undiagnosed infection declined from 22 to 8% [16].

GBM with undiagnosed HIV are believed to contribute disproportionately to HIV transmission in Australia [10] and have been estimated to account for the majority of new infections in some high-income countries [17–19]. In the absence of reliable national estimates, we set out to generate a more comprehensive measure of undiagnosed HIV among Australian GBM and to assess the characteristics of those with undiagnosed infection.

## Methods

### Participants and procedures

Participants were recruited through routine behavioural surveillance (the Gay Community Periodic Surveys or GCPS) conducted in six Australian cities from November 2013 to November 2014. Recruitment occurred during local gay festivals, for example Sydney's Mardi Gras. Time-location sampling was conducted by trained staff at gay festival events, bars, clinics and sex-on-premises venues. Eligible participants were male, aged 18 years or older, reported sex with another man in the previous five years and/or identified as gay/bisexual. Consenting participants self-completed an anonymous questionnaire. Details of GCPS procedures have been published elsewhere [5,20].

For the present study (Community-Based Study of Undiagnosed HIV and Testing or COUNT), GCPS participants were asked to participate in an additional study in which HIV prevalence would be measured using a biological (oral fluid) sample and test results matched with questionnaires. Immediately prior to recruitment in each city, advertising was conducted in gay media and venues explaining that the study was occurring and that we wanted “to find out how many gay and bisexual men have HIV, including men who don't currently know they're infected.” Due to resource and feasibility constraints, COUNT recruitment took place during about three-quarters of GCPS shifts; clinics were excluded. If willing, men were referred to additional trained staff who explained the study. Men who agreed to participate then completed a consent form and provided contact details if they wished to receive their test results. Participants could participate anonymously (no test results) or confidentially (test results provided). Oral fluid specimens were collected using the OraSure oral specimen collection device. To link bio-behavioural data and facilitate test result delivery, field staff labelled the participant's specimen, questionnaire and consent form with a unique identifier and the participant's date of birth.

Specimens were sent to the National Serology Reference Laboratory for testing using an anti-HIV-1 IgG antibody capture enzyme-linked immunosorbent assay [21]. All reactive and indeterminate samples were retested and confirmed by Western blot. Test results, questionnaires and consent forms were sent to the Centre for Social Research in Health for data processing and secure storage.

Delivery of test results was undertaken by the Burnet Institute. Result notifications were primarily delivered by mobile phone text message containing a link to a secure website, unless participants opted to be called or emailed. Participants with non-reactive results were notified that their result was negative (not mentioning HIV) and given a link to a secure website providing information about the study, the test result, its window period and support contacts. Participants who had reactive test results and whose self-reported HIV status indicated a previous diagnosis were either sent a message that their result was positive and a link to a secure website or they were asked to call the free study telephone line (in smaller jurisdictions, we spoke to all participants with reactive results who had provided contact details). For previously diagnosed men, the secure website provided information about the study, the test result and support contacts. Participants with indeterminate or reactive results that suggested previously undiagnosed HIV were sent a message that their results were ready and asked to call the study telephone line. If they did not call within 48 hours, they were called repeatedly until contacted. Trained staff handled all calls, provided support and facilitated appointments for confirmatory serology at local clinics. Participants undergoing confirmatory testing were followed up, subject to their consent.

The COUNT study was approved by the ethics committees of UNSW Australia, the community organization AIDS Council of New South Wales (ACON), the Australian Capital Territory Department of Health and the Victorian AIDS Council. ACON and the study reference group, which included the Australian Federation of AIDS Organisations and the National Association of People with HIV Australia, provided advice about the acceptability of the study to GBM.

### Measures

To describe the sample and assess associations with undiagnosed HIV, we assessed sociodemographic characteristics, sexual practices and relationships with men in the previous six months, recent HIV and sexual health testing history and drug use. Details of these measures have been published [20]. The primary outcome variable for this analysis was the participant's HIV status (HIV negative, previously diagnosed HIV positive, previously undiagnosed HIV positive), calculated by comparing the participant's questionnaire data (primarily self-reported HIV status) and laboratory HIV test results. We matched HIV test results with questionnaire data and resolved any discrepancies by speaking to the participant, provided they had consented to be contacted, or by referring to their questionnaire (self-reported HIV status, year of HIV diagnosis, HIV treatment, attending HIV care, viral load and CD4 cell count).

### Data analysis

To assess recruitment biases, COUNT and GCPS participants were compared with multivariate logistic regression, controlling for recruitment location, demographic characteristics, sexual practices, HIV testing history, self-reported HIV status and drug use. We report the prevalence of HIV and undiagnosed infection by recruitment arm (anonymous/confidential) and location. We calculated undiagnosed HIV as a proportion of HIV-positive test results and as a proportion of men who indicated they were HIV negative, untested or of unknown status when they entered the study. Using estimates of the size of the Australian GBM population [2], we adjusted the raw data to produce weighted national estimates of HIV prevalence and undiagnosed HIV. Men with previously undiagnosed HIV were compared with confirmed HIV-negative men and previously diagnosed men using logistic regression. Unadjusted odds ratios and 95% confidence intervals (CIs) were calculated (due to the small number of undiagnosed men we could not perform a multivariate analysis). Analyses were conducted using SPSS Version 22. Statistical significance was set at *p*<0.05.

## Results

During the study recruitment period, 7291 men were recruited into the GCPS in six cities. The participation rate across cities ranged from 65 to 91% (calculated as the percentage of eligible men who agreed to take part, recorded by recruitment staff during each shift). A total of 3085 men were referred from GCPS field staff and consented to take part in COUNT (42.3% of GCPS participants). Participants were excluded if their questionnaire, consent form or oral fluid sample was missing, could not be matched (due to a missing or illegible label) or because their sample was unviable for testing. This left 3071 participants in the current analysis. Of these participants, 842 (27.4%) took part anonymously (did not receive their test results) and 2229 (72.6%) participated confidentially (received their results).

A comparison of COUNT (*n*=3071) and GCPS-only participants (*n*=2961) at locations where COUNT recruitment took place found that the two samples had generally similar characteristics and behaviours including education level, sexual identity, number of recent male partners, condomless sex with casual male partners, use of non-condom-based risk reduction strategies, engagement in group sex and use of party drugs for sex (analyses not shown). COUNT participants were less likely to self-report being HIV positive (6.3% vs. 9.9%; adjusted odds ratio [AOR]=0.66, 95% CI=0.53 to 0.81). They were also less likely to be aged 30 or over (AOR=0.71, 95% CI=0.63 to 0.80) or to have been tested for HIV in the previous six months (AOR=0.79, 95% CI=0.70 to 0.90). COUNT participants were more likely to be Anglo-Australian (AOR=1.13, 95% CI=1.01 to 1.27), to have been recruited at a gay festival event (AOR=2.06, 95% CI=1.85 to 2.31) and to report condomless anal intercourse with a regular male partner in the previous six months (AOR=1.42, 95% CI=1.24 to 1.62).

On enrolment, 2490 of 3071 COUNT participants reported that they were HIV negative, 196 HIV positive and 385 as untested/unknown HIV status. After matching with test results and resolving discrepancies, 2858 men (93.1%) were confirmed as HIV negative, 194 as previously diagnosed HIV positive (6.3%) and 19 (0.6%) as previously undiagnosed HIV positive. Twelve of the previously undiagnosed men received their test results.

### Participant characteristics

Participant characteristics can be seen in Table 1. The mean age of the sample was 36 years. The majority were recruited from gay festival events and over a third from gay bars and sex-on-premises venues. The Internet and mobile apps were the most common ways to meet male sex partners. The majority of the sample were Anglo-Australian, university-educated and in full-time employment. The majority self-identified as gay, and over one-third said most of their friends were gay and they spent a lot of time with gay men. A small proportion (6%) said they had a regular HIV-positive partner. One-fifth had had more than ten male sex partners in the past six months, over one-third said they had had any condomless anal intercourse with regular male partners and just under one-quarter with casual male partners. Slightly less than one-third reported any group sex in the past six months, and over one-third had disclosed their perceived HIV status during casual sex. Of HIV risk reduction strategies frequently used during anal intercourse with casual partners, the most common strategy was condoms followed by serosorting (matching perceived HIV status before condomless sex). Small proportions (<5%) reported the frequent use of strategic positioning, undetectable viral load and withdrawal before ejaculation as risk reduction strategies during condomless sex with casual partners [22]. Almost half the sample reported that they had been tested for HIV in the previous six months, over half had been tested for sexually transmissible infections (STIs) and one in seven had been diagnosed with an STI other than HIV. Very few men reported being prescribed post-exposure prophylaxis (PEP) or taking antiretroviral drugs as pre-exposure prophylaxis (PrEP). In terms of drug use in the past six months, nearly one-fifth of the sample reported using party drugs for sex and nearly 1 in 20 reported injecting drug use. Commonly used drugs included amyl nitrite, cannabis/marijuana and ecstasy.

**Table 1:** Participant characteristics stratified by HIV status and associations with undiagnosed HIV

		HIV status according to test result			Associations with undiagnosed HIV	
			
	Whole sample (*n*=3071)	HIV negative (*n*=2858)	Previously diagnosed HIV positive (*n*=194)	Previously undiagnosed HIV positive (*n*=19)	Reference group: HIV negative	Reference group: diagnosed HIV positive
						
	%	%	%	%	Odds ratio (95% CI)	Odds ratio (95% CI)
Age (M, SD)	35.6 (12.2)	35.1 (12.1)	43.3 (11.0)	32.6 (8.1)	0.98 (0.94–1.02)	0.89 (0.84–0.95)***
Recruitment arm						
Anonymous	27.4	24.0	76.3	36.8	1.00	1.00
Confidential	72.6	76.0	23.7	63.2	0.54 (0.21–1.38)	5.52 (2.05–14.83)***
Recruitment location						
Gay festival event	63.0	63.2	60.8	63.2	1.00	1.00
Gay bar or sex-on-premises venue	37.0	36.8	39.2	36.8	1.00 (0.39–2.55)	0.91 (0.34–2.40)
How men met male sex partners in past six months						
Internet	36.2	35.4	47.4	42.1	1.33 (0.53–3.31)	0.81 (0.31–2.09)
Mobile app (e.g. Grindr, Scruff)	45.6	45.6	43.8	68.4	2.59 (0.98–6.83)	2.78 (1.01–7.61)*
Gay bar	27.5	27.5	25.3	36.8	1.54 (0.60–3.91)	1.73 (0.64–4.63)
Sex-on-premises venue	28.9	27.5	44.8	63.2	4.51 (1.77–11.50)**	2.11 (0.80–5.58)
Anglo-Australian background	69.4	69.1	74.2	68.4	0.97 (0.37–2.56)	0.75 (0.27–2.09)
Completed university degree	52.4	52.7	50.0	42.1	0.65 (0.26–1.63)	0.73 (0.28–1.89)
Full-time employed	63.6	64.1	57.2	52.6	0.62 (0.25–1.54)	0.83 (0.32–2.14)
Gay-identified	89.1	88.7	94.3	100.0	–	–
Most or all friends are gay men	43.3	42.2	59.3	47.4	1.23 (0.50–3.04)	0.62 (0.24–1.59)
A lot of free time spent with gay men	38.1	37.5	47.9	15.8	0.31 (0.09–1.07)	0.20 (0.06–0.72)**
Had HIV-positive regular partner (at time of survey)	4.1	2.8	23.7	0.0	–	–
More than 10 male sex partners in past six months	19.5	18.5	34.0	21.1	1.18 (0.39–3.56)	0.52 (0.17–1.62)
Any condomless anal intercourse in past six months						
With regular partners~	40.4	40.4	41.2	36.8	0.86 (0.34–2.19)	0.83 (0.31–2.20)
With casual partners~	23.1	21.6	42.3	47.4	3.27 (1.32–8.08)**	1.23 (0.48–3.16)
Any group sex in past six months	30.0	28.9	45.4	26.3	0.88 (0.31–2.44)	0.43 (0.15–1.24)
Disclosed HIV status to some/all casual partners in past six months	38.3	36.7	61.9	36.8	1.01 (0.40–2.57)	0.36 (0.14–0.95)*
FrequentΛ use of risk reduction strategies during anal intercourse with casual partners in past six months						
Condoms	24.1	24.2	22.2	36.8	1.83 (0.72–4.67)	2.05 (0.76–5.52)
Serosorting	12.0	11.0	26.3	21.1	2.15 (0.71–6.53)	0.75 (0.24–2.36)
Strategic positioning	4.9	4.7	8.2	5.3	1.12 (0.15–8.46)	0.62 (0.08–4.94)
Undetectable viral load	3.8	2.0	30.9	5.3	2.78 (0.36–21.19)	0.12 (0.02–0.95)*
Withdrawal before ejaculation	4.8	4.7	5.7	5.3	1.13 (0.15–8.52)	0.92 (0.11–7.57)
HIV test in past six months	45.3	45.3	–	47.4	1.09 (0.44–2.69)	–
Any STI test in past six months (excluding blood test)	60.7	58.9	86.6	68.4	1.51 (0.57–3.99)	0.34 (0.12–0.96)*
STI diagnosis in past six months	13.3	12.2	27.8	15.8	1.34 (0.39–4.63)	0.49 (0.14–1.74)
Prescribed PEP in past six months	3.1	3.1	–	5.3	1.75 (0.23–13.25)	–
Taken antiretroviral drugs as PrEP in past six months	1.5	1.5	–	10.5	7.89 (1.77–35.23)**	–
Used party drugs for sex in past six months	19.0	17.9	32.0	42.1	3.32 (1.33–8.31)**	1.55 (0.59–4.04)
Injected any drugs in past six months	4.1	3.1	18.0	21.1	8.39 (2.73–25.81)***	1.21 (0.38–3.87)
Drugs used in past six months						
Amyl nitrite (poppers)	37.3	36.2	51.5	63.2	3.02 (1.19–7.70)*	1.61 (0.61–4.27)
Cannabis/marijuana	30.7	30.2	38.7	31.6	1.07 (0.40–2.82)	0.73 (0.27–2.01)
Crystal methamphetamine (crystal, ice)	11.1	9.9	26.8	26.3	3.25 (1.16–9.09)*	0.98 (0.33–2.84)
Ecstasy	20.8	20.8	18.6	36.8	2.22 (0.87–5.66)	2.56 (0.94–6.69)
Erectile dysfunction medication	17.3	16.0	35.6	26.3	1.88 (0.67–5.25)	0.65 (0.22–1.87)
Gamma hydroxybutyrate	6.7	6.0	15.5	26.3	5.61 (2.00–15.76)***	1.95 (0.065–5.82)

CI, confidence interval; M, mean; SD, standard deviation;

* *p*<0.05;

** *p*<0.01;

*** *p*<0.001;

~ among the whole sample, not just men with regular or casual partners; STI, sexually transmissible infection; PEP, post-exposure prophylaxis; PrEP, pre-exposure prophylaxis;

Λ frequently, often or always using the strategy during anal intercourse.

### Prevalence estimates

Prevalence estimates for HIV and undiagnosed infection are shown in Table 2. HIV prevalence ranged from 4.7% in Canberra to 8.8% in Brisbane, with a national estimate of 6.9% HIV positive. HIV prevalence was higher in the anonymous arm compared to the confidential arm, with previously diagnosed men being more likely to participate anonymously and not receive their test results. HIV prevalence was similar among men recruited at venues and events. As a proportion of confirmed HIV-positive results, undiagnosed HIV ranged from 0% in Canberra to 19.0% in Perth, with a national estimate of 8.9% undiagnosed. Because previously diagnosed men largely participated in the anonymous arm, undiagnosed HIV as a proportion of HIV-positive cases was much higher in the confidential arm than the anonymous arm (20.7% vs. 4.5%). The prevalence of undiagnosed HIV was similar among men recruited at gay venues and events. As a proportion of men who reported they were HIV negative, untested or of unknown status, undiagnosed HIV ranged from 0% in Canberra to 1.4% in Perth, with a national estimate of 0.7% undiagnosed.

After adjusting the raw data using estimates of the size of the gay and bisexual male population in each state and territory (2), the weighted national estimate of HIV prevalence was 7.2% (95% CI 6.3 to 8.1). The weighted national estimates of undiagnosed HIV were 9.1% as a proportion of confirmed HIV-positive results (95% CI 6.0 to 13.6%) and 0.7% as a proportion of men who believed they were HIV negative, were untested or had unknown status on enrolment (95% CI 0.5 to 1.1%).

**Table 2:** Prevalence estimates of HIV and undiagnosed infection by recruitment arm and location

			Undiagnosed HIV			
			
Recruitment arm or location (number of participants)	HIV-positive participants (confirmed by testing)		As proportion of HIV-positive participants (confirmed by testing)		As proportion of HIV-negative and unknown status participants (self-reported)	
		
	*n* (%)	95% CI	*n* (%)	95% CI	*n* (%)	95% CI
Anonymous^1^ (*n*=842)	155 (18.4)	15.9–21.2	7 (4.5)	2.2–9.0	7 (1.0)	0.5–2.1
Confidential^2^ (*n*=2229)	58 (2.6)	2.0–3.3	12 (20.7)	12.3–32.8	12 (0.5)	0.3–1.0
Adelaide (*n*=369)	20 (5.4)	3.5–8.2	1 (5.0)	0.9–23.6	1 (0.3)	0.1–1.6
Brisbane (*n*=373)	33 (8.8)	6.4–12.2	2 (6.1)	1.7–19.6	2 (0.6)	0.2–2.1
Canberra (*n*=86)	4 (4.7)	1.8–11.4	0 (0.0)	0.0–49.0	0 (0.0)	0.0–4.5
Melbourne (*n*=933)	70 (7.0)	5.6–8.8	5 (7.1)	3.1–15.7	5 (0.5)	0.2–1.3
Perth (*n*=306)	21 (6.9)	4.5–10.3	4 (19.0)	7.7–40.0	4 (1.4)	0.5–3.5
Sydney (*n*=944)	65 (6.9)	5.4–8.7	7 (10.8)	5.3–20.6	7 (0.8)	0.4–1.6
Gay bar or sex-on-premises venue (*n*=1135)	83 (7.3)	5.9–9.0	7 (8.4)	4.1–16.4	7 (0.7)	0.3–1.4
Gay festival event (*n*=1936)	130 (6.7)	5.7–7.9	12 (9.2)	5.4–15.4	12 (0.7)	0.4–1.2
Total (*n*=3071)	213 (6.9)	6.0–7.8	19 (8.9)	5.8–13.5	19 (0.7)	0.4–1.0

CI, confidence interval;

1 no test results given to participants;

2 test results provided.

### Associations with undiagnosed HIV

Participant characteristics stratified by HIV status and associations with undiagnosed HIV can be seen in Table 1. There were no statistically significant sociodemographic differences between previously undiagnosed HIV-positive men and confirmed HIV-negative men. Recruitment arm or location, the number of male partners, condomless sex with regular partners, engagement in group sex, HIV disclosure, the use of different risk reduction strategies or HIV/STI testing history did not distinguish between these two groups. Compared with HIV-negative men, previously undiagnosed HIV-positive men had significantly higher odds of meeting partners at sex-on-premises venues, reporting condomless sex with casual partners, the use of antiretrovirals as PrEP, the use of party drugs for sex, any injecting drug use and the use of amyl nitrite, crystal methamphetamine and gamma hydroxybutyrate (GHB). The two undiagnosed men who reported PrEP use indicated they had been prescribed daily medication; both participated anonymously so we could not find out more about their use.

Previously undiagnosed men were younger than previously diagnosed men and were less likely to spend time with gay men. There were no other sociodemographic differences between these two groups. Previously undiagnosed men were more likely than diagnosed men to use mobile apps to meet sex partners and less likely than diagnosed men to disclose their HIV status to casual partners, to use undetectable viral load as a risk reduction strategy during condomless sex or to have been tested for STIs in the past six months. Undiagnosed men engaged in condomless sex, group sex and drug use (including injecting) at similar levels to previously diagnosed men.

To clarify how and when undiagnosed men (*n*=19) may have been exposed to HIV, we analyzed their recent sexual practices and HIV testing history. Four men reported condomless sex with both casual and regular male partners in the previous six months, five men reported condomless sex with casual partners only, three with regular partners only and seven did not report condomless sex in the previous six months. Nine men reported testing for HIV in the previous six months, two men between 6 and 12 months ago, three men one to two years ago and two men over two years ago. One man had never been tested for HIV and two men did not report their testing history.

## Discussion

We have conducted the largest study to date of HIV prevalence and undiagnosed infection among Australian GBM. We found a national HIV prevalence of 7% in this population and determined that 9% of HIV-positive men were undiagnosed. There was a wide range of undiagnosed infection (0 to 19% of HIV-positive men) across cities and wide CIs for estimates in cities with smaller sample sizes. Adjusting the estimates to allow for the size of the GBM population in each state and territory did not noticeably change the estimates. Our estimate of HIV prevalence falls between the self-reported 5% found in national household samples [2,3] and the 10% reported by GBM in larger cities [4,5]. Our estimate of undiagnosed HIV is considerably lower than that found in previous studies conducted at gay venues in Melbourne and Queensland [6,7] and slightly lower than that used in recent modelling of the Australian epidemic [8,9]. Internationally, our results indicate that undiagnosed HIV among Australian GBM is among the lowest seen in high-income countries with concentrated epidemics among GBM [11–15]. Only San Francisco and Vancouver appear to have similarly low levels of undiagnosed infection [15,16].

There are a number of possible explanations for why we found a lower level of undiagnosed HIV in this study compared with other Australian and international research. An optimistic interpretation of our results, which we cannot prove or disprove, is that the gradual increase in the frequency of HIV testing by GBM over the last decade has resulted in a low level of undiagnosed infection [4,7,23]; we note that nearly half of all participants in the study reported an HIV test within the previous six months. A more pessimistic interpretation would be that we failed to recruit those men who were most likely to have undiagnosed HIV. We targeted a broader range of recruitment sites than studies solely focused on gay venues, and therefore it is possible that we recruited proportionally fewer men at high risk of HIV. However, the level of undiagnosed infection we found was similar among men recruited at gay venues and community events. It is possible that the offer of HIV test results may have dissuaded “at risk” men from participating, in contrast to completely anonymous studies [6,7], although we note that over 60% of previously undiagnosed men in our study opted to receive their test results. We appear to have under-recruited previously diagnosed HIV-positive men in the larger cities, compared to the prevalence of HIV found in routine behavioural surveillance [4,5]. The offer of HIV test results may have resulted in previously diagnosed men perceiving little benefit from their participation (recruitment staff reported that openly HIV-positive men were generally supportive, but occasionally expressed the view that the study was of limited relevance to them). The effect of under-recruiting previously diagnosed men can be seen most clearly in the confidential arm of the study, in which the proportion of undiagnosed cases over the HIV-positive denominator appears disproportionately large. For this reason, we also reported undiagnosed HIV as a proportion of men who self-reported an HIV-negative or untested status on enrolment. However, despite its limitations, we believe our estimate of undiagnosed HIV among Australian GBM is robust, including a broad range of GBM from across the country.

Our study found that previously undiagnosed men were generally more likely than HIV-negative men (and as likely as previously diagnosed men) to report practices associated with sex- and drug-based socializing and “intensive sex partying” [24,25]: having sex at sex venues, condomless sex with casual male partners, the use of party drugs for sex, injecting drug use and the use of amyl nitrite, crystal methamphetamine and GHB. Our findings suggest that men who engage in sex-related drug use remain at elevated risk of HIV in Australia [26,27] and that there is a continuing need for HIV prevention and harm reduction programmes for these men. Access to clinically supervised PrEP appears warranted, given that two undiagnosed men reported being prescribed PrEP during a period when it was not formally available in Australia. It is possible they may have confused PrEP with PEP (which was available). Using antiretrovirals did not ultimately protect them from HIV, which raises questions about how long and consistently they used the drugs and the degree of clinical support they received. Compared with HIV-negative men, a similar proportion of previously undiagnosed men reported testing for HIV in the previous six months, which suggests that they are not averse to testing but may not test as often as recommended. Australian guidelines recommend that GBM at high risk of HIV should test up to four times a year [28]. Previously undiagnosed men were more likely to report a recent STI test than a recent HIV test, suggesting that opportunities for comprehensive sexual health screening continue to be missed when GBM present for care [4].

## Conclusions

Our study found a relatively low level of undiagnosed HIV among GBM in six Australian cities. The results suggest that recent modelling of the Australian epidemic may have slightly overestimated the burden of undiagnosed infection among GBM in metropolitan areas [8,9]. The profile of men with undiagnosed infection in our study highlights the ongoing need for HIV prevention and harm reduction programmes for GBM who have drug-based sex and condomless sex with casual partners.
